# Supplementary material for: An open-source framework for large-scale, flexible evaluation of biomedical text mining systems
Source: J Biomed Discov Collab. 2008 Jan 29;3:1. doi: 10.1186/1747-5333-3-1 (PMC2276192; doi:10.1186/1747-5333-3-1)
Supplement: Additional file 1 — Evaluation platform source code. The additional file contains the software evaluation framework discussed in this paper. The most current release can be downloaded from SourceForge.net [1]. See the accompanying README file for instructions on installation and use. Also included in the distribution is a collection of UIMA wrappers for some commonly used BioNLP tools and annotated corpora. [file 1747-5333-3-1-S1.zip › bionlp_uima_repository-1.0.1/docs/api/edu/uchsc/ccp/util/nlp/parser/corpus/CorpusParser.html]

CorpusParser (Center for Computational Pharmacology Util/UIMA API)


|  |  |  |
| --- | --- | --- |
| **Overview** \| **Package** \| **Class** \| **Tree** \| **Deprecated** \| **Index** \| **Help** | |  |
| **PREV CLASS**   **NEXT CLASS** | **FRAMES**    **NO FRAMES**     **All Classes** |  |
| SUMMARY: NESTED \| FIELD \| CONSTR \| METHOD | DETAIL: FIELD \| CONSTR \| METHOD |  |


---


## edu.uchsc.ccp.util.nlp.parser.corpus Class CorpusParser

```
java.lang.Object
  edu.uchsc.ccp.util.nlp.parser.corpus.CorpusParser
```

**All Implemented Interfaces:**: Iterator

**Direct Known Subclasses:**: Bio1CorpusParser, BioIECorpusParser, GeniaCorpusParser, IProLinkCorpus1Parser, IProLinkCorpus2Parser, TexasCorpusParser, YapexCorpusParser

---

``` public abstract class CorpusParser extends Object implements Iterator ```

This is an abstract class for a corpus parser. It follows the `Iterator` interface, and should return a `GenericDocument` object
for each document in the corpus being parsed.

**Author:**
:   William A Baumgartner, Jr.

---

**Constructor Summary**

`CorpusParser()`


| **Method Summary** | |
| --- | --- |
| `abstract  void` | `close()` |
| `protected static TextAnnotation` | `createTextAnnotation(String documentID, int documentCollectionID, int spanStart, int spanEnd, String classMentionType, String coveredText, Annotator annotator, AnnotationSet annotationSet, int annotationID)`             Creates a new TextAnnotation |
| `protected  void` | `error(String message)` |
| `abstract  boolean` | `hasNext()` |
| `abstract  GenericDocument` | `next()` |
| `void` | `remove()` |
| `protected  void` | `warn(String message)` |

**Methods inherited from class java.lang.Object**

`clone, equals, finalize, getClass, hashCode, notify, notifyAll, toString, wait, wait, wait`

**Constructor Detail**

### CorpusParser

```
public CorpusParser()
```


**Method Detail**

### hasNext

```
public abstract boolean hasNext()
```

:   **Specified by:**: `hasNext` in interface `Iterator`

---


### next

```
public abstract GenericDocument next()
```

:   **Specified by:**: `next` in interface `Iterator`

---


### close

```
public abstract void close()
                    throws IOException
```

:   **Throws:**: `IOException`

---


### remove

```
public void remove()
```

:   **Specified by:**: `remove` in interface `Iterator`

---


### error

```
protected void error(String message)
```

---


### warn

```
protected void warn(String message)
```

---


### createTextAnnotation

```
protected static TextAnnotation createTextAnnotation(String documentID,
                                                     int documentCollectionID,
                                                     int spanStart,
                                                     int spanEnd,
                                                     String classMentionType,
                                                     String coveredText,
                                                     Annotator annotator,
                                                     AnnotationSet annotationSet,
                                                     int annotationID)
```

:   Creates a new TextAnnotation

    :   **Parameters:**: `documentID` -: `documentCollectionID` -: `spanStart` -: `spanEnd` -: `classMentionType` -: `coveredText` -: `annotator` -: `annotationSet` - **Returns:**


---


|  |  |  |
| --- | --- | --- |
| **Overview** \| **Package** \| **Class** \| **Tree** \| **Deprecated** \| **Index** \| **Help** | |  |
| **PREV CLASS**   **NEXT CLASS** | **FRAMES**    **NO FRAMES**     **All Classes** |  |
| SUMMARY: NESTED \| FIELD \| CONSTR \| METHOD | DETAIL: FIELD \| CONSTR \| METHOD |  |


---
